# Multi-aspect testing and ranking inference to quantify dimorphism in the cytoarchitecture of cerebellum of male, female and intersex individuals: a model applied to bovine brains

**DOI:** 10.1007/s00429-020-02147-x

**Published:** 2020-09-28

**Authors:** L. Corain, E. Grisan, J.-M. Graïc, R. Carvajal-Schiaffino, B. Cozzi, A. Peruffo

**Affiliations:** 1 Department of Management and Engineering, University of Padova, 36100 Vicenza, VI Italy; 2 Department of Information Engineering, University of Padova, 35131 Padua, PD Italy; 3 School of Engineering, London South Bank University, London, SE1 0AA UK; 4 Department of Comparative Biomedicine and Food Science, University of Padova, Viale dell’Università 16, 35020 Legnaro, PD Italy; 5 Department of Mathematics and Computer Science, University of Santiago de Chile, Santiago, Chile

**Keywords:** Brain dimorphism, Cerebellum, Cytoarchitecture morphometrics, Image analysis, Multi-aspect analysis in neuroanatomy

## Abstract

The dimorphism among male, female and freemartin intersex bovines, focusing on the vermal lobules VIII and IX, was analyzed using a novel data analytics approach to quantify morphometric differences in the cytoarchitecture of digitalized sections of the cerebellum. This methodology consists of multivariate and multi-aspect testing for cytoarchitecture-ranking, based on neuronal cell complexity among populations defined by factors, such as sex, age or pathology. In this context, we computed a set of shape descriptors of the neural cell morphology, categorized them into three domains named size, regularity and density, respectively. The output and results of our methodology are multivariate in nature, allowing an in-depth analysis of the cytoarchitectonic organization and morphology of cells. Interestingly, the Purkinje neurons and the underlying granule cells revealed the same morphological pattern: female possessed larger, denser and more irregular neurons than males. In the Freemartin, Purkinje neurons showed an intermediate setting between males and females, while the granule cells were the largest, most regular and dense. This methodology could be a powerful instrument to carry out morphometric analysis providing robust bases for objective tissue screening, especially in the field of neurodegenerative pathologies.

**Electronic supplementary material:**

The online version of this article (10.1007/s00429-020-02147-x) contains supplementary material, which is available to authorized users.

## Introduction

### Morphometric data analytics

The concept of morphometrics was introduced in the early 1900s, but it was not until the 1980s that researchers started to use tools for the morphological analysis of cells and the identification of phenotypes. Since then, morphometric descriptors and tools were employed for the quantitative analysis of cell structure, and relevant geometrical features of the cell were, thus, defined by proper objective parameters. These latter descriptors quantity and typify certain cellular attributes (Pincus and Theriot 2007), provide a powerful tool in histological analysis and allow unbiased comparisons among diverse cell types (Thurner et al. 2005; Lobo et al. 2016). The reliability of image-based cellular studies increased as researchers translated qualitative differences into quantitative measurements, establishing an objective approach to cell shape (Pasqualato et al. 2012). These studies provided important steps forward to develop suitable statistical procedures for the analyses of morphological data. However, a method that considers all morphometric descriptors in one analysis could help provide a complete view of the tissue architecture. To date, the analysis of the cellular morphology in the brain cytoarchitecture is still a challenge, and there is an open quest to translate the qualitative differences observed microscopically into quantitative measurements (Lobo et al. 2016).

Cell shape is a powerful indicator of neural cells’ functions in health and pathology (Devlin and Poldrack 2007; Amunts et al. 2007b). To this effect, morphometric parameters including neuronal size, density or volume may compose a multi-scale view to quantify the distribution of specific neurons (Silvestri et al. 2015) and develop new methods to systematically extract useful information from neuroanatomical data (DuBois Bowman et al. 2007; Ozaki 2014; Graïc et al. 2020, in press). The need for advanced statistical methods and modern techniques for image analysis of neural cells led to several specific studies in our laboratory (Montelli et al. 2017; Cozzi et al. 2017; Graïc et al. 2018).

### Sexual dimorphism and the cerebellar cortex

Several studies demonstrated the existence of sexual differences in the organization of the mammalian brain, functionally relevant for certain cerebral functions in health and pathology (McCarthy et al. 2012; Marocco and McEwen 2016; De Vries and Forger 2015; McEwen and Milner 2017). However, the differences are subtle, involve regional variability, and are still controversial (Chekroud et al. 2016; Del Giudice et al. 2016; Ingalhalikar et al. 2014; Joel and Tarrasch 2014).

The laminar organization and cytoarchitecture of the adult cerebellar cortex are highly constant across mammals (Voogd and Glickstein 1998; Apps and Hawkes 2009; Sultan and Glickstein 2007; Jacobs et al. 2014), although recent anatomical, physiological and genetic evidence indicate the presence of regional differences, including variations in cell type, morphology and expression markers (Cerminara et al. 2015). However, while the general topography of functional zones in the cerebellum is widely accepted and gender differences in behaviors are documented (Jazin et al. 2010; Cohen-Bendahan et al. 2005), structural dimorphism and asymmetry have been poorly described and results are still subject to debate (Fan et al. 2010). In humans, literature reports gender differences in cerebellar mass (Dimitrova et al. 2006; Ruigrok et al. 2014; Weier et al. 2014), lobular volume (Diedrichsen et al. 2009; Dimitrova et al. 2006; Raz et al. 1998, 2001; Rhyu et al. 1999; Tiemeier et al. 2010; Weier et al. 2014; Steele and Chakravarty 2018), neuroendocrine response (Dean and Mccarthy 2008; Abel et al. 2011), and dimensions of Purkinje cells (Müller and Heinsen 1984). A relationship between lobular volumes and behavior has been established, (Bernard et al. 2015; Bernard and Seidler 2013), but studies providing an exhaustive account of how structure may differ between sexes are lacking.

The cerebellum is involved in neurodevelopmental diseases including autism spectrum disorders (ASD), cerebellar ataxia and embryonic tumor medulloblastoma (Hampson and Blatt 2015; Mercer et al. 2016; Marzban et al. 2018). The incidence of ASD is much higher in men than in women (Halladay et al. 2015), and structural differences have been consistently reported in their cerebellum (Allen et al. 2004; Courchesne and Allen 1997; Fatemi et al. 2012). Finally, an exploratory study examining the cerebellar vermis in humans with schizophrenia using a quantitative volumetric approach has shown a greater reduction in vermis volume in males than females (Womer et al. 2016).

There is increasing attention brought onto the development of new animal models to understand the anatomical and genetic basis of neurodegenerative disorders (McGonigle and Ruggeri 2014), and the domestic, fairly standardized *Bos taurus* can be a proper candidate (Peruffo et al. 2014). Their gestation period (41 weeks) is comparable to the human pregnancy (38–40 weeks), and their brain is large and highly convoluted (Ballarin et al. 2016). The key factor in favor of this model is that bovine frequently shows naturally occurring intersex calves, due to the freemartin syndrome. This condition occurs following the formation of vascular connections between the placentas of heterosexual twin fetuses and disturbs the sex differentiation of the female twin via the anti-Müllerian hormone production (Rota et al. 2002; Cabianca et al. 2007). Visible consequences on freemartin heifers include body masculinization (Gregory et al. 1996), dramatic changes in the reproductive tract and failure to enter estrus (Marcum 1974; Long 1990; Padula 2005). In this context, the intersex bovine freemartin offers an interesting model to study sex differences of the brain and development in translational medicine (Graïc et al. 2018). Furthermore, a previous in vitro study performed on this species in our laboratory reported that granule cells of the female cerebellum showed significantly larger morphological values than the corresponding male elements (Montelli et al. 2017).

Since the cerebellum offers a good model to develop computational statistical approaches to the study of single cell morphology, we studied the structure of vermal lobules VIII and IX of male, female and intersex freemartins bovines. The present study aims at providing clarification on controversial results in sex-related cerebellar differences while acknowledging the freemartin syndrome as a valuable intersex animal model. In addition, this multivariate and multi-aspect method can be extended to study virtually any brain region, providing a robust base for tissue screening, including for the presence of neurodegenerative features.

## Materials and methods

### Tissue sampling

A series of 28 adult bovine brains (10 males, 10 females and 8 freemartins, all 24 months old), were obtained from local abattoirs in the Veneto region. Animals were treated according to the present European Community Council directive concerning animal welfare during the commercial slaughtering process and were constantly monitored under mandatory official veterinary medical care. The cerebella were collected under sterile conditions and fixed by immersion in phosphate-buffered formaldehyde 4% for 1 month. From each cerebellum, the lobules VIII and IX, classical paleocerebellar lobules located at the postero-inferior part of the vermis, were sampled, re-immersed in buffered formalin, then washed in phosphate saline buffer (PBS) 0.1 M, pH 7.4 and processed for paraffin embedding.

### Nissl staining

The lobules VIII and IX of each specimen were cut into 8-µm-thick parasagittal sections. For each cerebellar sample, one section every five was stained (a total of 10 slides per individual per sex). Sections were stained following a standard Nissl protocol: sections were deparaffinized in xylene for 3 × 5 min, followed by a hydration series in graded alcohols for 3 min each. After 3 min in distilled water, sections were stained in 0.1% cresyl violet solution for 10 min at 57 °C. Sections were then differentiated in 95% alcohol for 20 min. After rinsing in distilled water, sections followed an ascending series of dehydration in graded alcohols for 3 min each, and finally 3 × 5 min in xylene. The sections were then covered with mounting medium and coverslip glass.

The most recent anatomical description of the bovine brain (Okamura 2002) contains illustrations of coronal sections including the main features of the subcortex. Additional details can be found in Yoshikawa (1968). The gross anatomy of the cerebellum was assessed using these references and from Voogd (1998) and Voogd and Glickstein (1998).

All the brains used in the present study were extracted with a post-mortem interval no longer than 4 h, and subsequently spent the same amount of time in formalin. The brains were then processed following the same paraffin-embedding, cutting and staining protocol, to obtain remarkably constant results. Moreover, each staining run contained female, male and freemartin sections.

### Image acquisition and automatic cell identification

Ten stained sections per subject were scanned with a semi-automated microscope equipment (D-Sight v2, Menarini Diagnostics, Italy) at a magnification of 40×, using constant lighting profiles.

Based on these digital images, the limits between layers were drawn independently by 3 neuroanatomists (AP, JMG, BC), each working autonomously using a raster image software (GNU Image Manipulation Program, Free Software Foundation, Inc.), and then compared until consensus was reached.

The complete analysis of the acquired images of cerebellar slices involves the detection and outline of tens of thousands of cells. This is not feasible by human annotation of the images, unless the procedure is carried out in small region of interest, potentially introducing bias in the procedure. To tackle the problem, we developed an automatic procedure (Grisan et al. 2018) that can process the images identifying the position and outline of most of the visible cells, taking care of the different sizes among the different cell populations, and at the same time addressing the packed and clustered appearance of cells in the different layers of the cerebellum, particularly in the granule cells layer.

The images were exported as Jpeg2000, resulting in a mean dimension of 42,000 × 42,000 pixels with a resolution of 0.5 μm per pixel. Each image was downsampled, to keep the computational burden low, to an equivalent resolution of 1 μm per pixel. The average target intensity was locked at 71% to ensure that the exposure was kept uniform while sampling.

The analyzed data consisted of information on tens of thousands individual neural cells. In a preliminary test, cells were localized within the three layers identified by the independent observers, and compared to the algorithm’s results.

Table 1 reports the performance of the proposed and competing algorithms in absolute numbers of detected cells (first column), wrong detections (second rows), and detected areas corresponding to multiple cells that were not separated (for details see Grisan et al. 2018).

**Table 1:** Algorithm comparison

Method	Detected cells (TP)	Non-cell detection (FP)	Remaining clusters
Al-Kofahi et al. (2010)	1837	2178	14
Lu et al. (2016)	2280	9226	94
Ram and Rodriguez (2016)	**3561**	7233	56
Proposed	3294	**488**	**20**

The quality of the detection performance of the algorithm was assessed based on its ability to correctly identify a cell (true positive, TP), to minimize the number of background. Please provide the subjects erroneously identified as cells (false positives, FP), and to correctly separate clusters of cells (remaining clusters). The proposed algorithm performed well both in terms of precision and recall, obtaining a *F*1-score of 0.87 on cerebellum Nissl-stained images.

Shortly (for additional details we refer to Grisan et al. 2018), a local space-varying threshold (Poletti et al. 2012) is applied to the image to separate the stained objects from the background, and from the local density of the foreground objects (mainly cells), a rough separation of the most densely (possibly with clustered and cluttered cells) and sparsest regions is obtained. Then, a small set of thresholds on the values of eccentricity, areas and solidity of the identified objects allows the identification of single small cells (limited area, high circularity and solidity), Purkinje cells (large area, high circularity, decreasing solidity with area), from possible clusters of cells.

All the objects that were appraised as a possible cluster undergo further analyses to disaggregate the individual cells that compose it. This is performed by modeling the intensity appearance of a cell as with 2-dimensional Gaussian shape. In case of clustered cells, this leads to a representation of the cluster as a mixture of Gaussian, with a number of modes corresponding to the number of cells composing the cluster. Hence, from the original image $I$, around each identified cluster, a sub-image ${I}_{\mathrm{clu}}$(*x*, *y*) is extracted. The sub-image intensities are assumed to be described by a bi-dimensional Gaussian mixture model (GMM) containing several modes $N$ equal to the number of the local maxima:

$$G\left(x,y;{c}_{i},{\Sigma }_{i}\right)={e}^{-0.5{(\left(x,y\right)-{c}_{i})}^{T}{{\Sigma }_{i}}^{-1}(\left(x,y\right)-{c}_{i})}$$

$$\mathrm{GMM}\left(x,y\right)=\sum_{i=1}^{N}{\alpha }_{i}G(x,y;{c}_{i},{\Sigma }_{i})$$

The parameters of the mixture of Gaussians are estimated by a non-linear least square fit to the sub-image data, and they provide both the center and dimension of the cells forming the cluster.

### Cell morphometric descriptors definition

Each identified cell is then described by a set of morphometric measures characterizing its shape and local relationship with surrounding cells. These measures can be broadly assigned to three domains: size, regularity and density. Size and regularity domain address cell morphology and are composed by classical measures on shapes. The density domain characterizes the context around each cell by counting the number of cells that are present within a radius of 50 µm or within 100 µm. See Table 2.

**Table 2:** Morphological domains and morphometric descriptors, along with their description and/or mathematical formula

Morphological domain	Morphometric descriptor	Description/mathematical formula
Size	Area	Area of the cell body expressed in μm^2^
	Perimeter	Total length of neural cell boundary expressed in μm
	Major axis length	Measure of the length of the major axis of the cell body expressed in μm
	Minor axis length	Measure of the length of the minor axis of the cell body expressed in μm
Regularity	Solidity	Proportion of pixels in the convex hull that are also in the region of the cell
	Extent	Area/(area of the bounding box)
	Inv.AR (1/AR)	Inverse of the aspect ratio, defined as (major axis length)/(minor axis length)
	Convex circularity	(4 × π × convex area)/(convex perimeter^2^)
Density	Ngb_50	No. of neighbor cells counted within a radius of 50 μm around a given cell
	Ngb_100	No. of neighbor cells counted within a radius of 100 μm around a given cell

Actual data were obtained using corresponding Matlab functions. Convex circularity was used instead of traditional circularity to avoid meaningless values

It is worth noting that for size-related morphometric measures, a natural positive correlation exists with the neuron’s soma size. For regularity-based descriptors, the larger they are, the more regular is the neuron. Notably, all regularity descriptors are dimensionless ratios bounded in the closed interval [0;1]. Finally, both density-related descriptors refer to the amount of neighbor cells present around a given cell.

We analyzed separately in each cortical layer the morphometric data (inference on location), and the related anatomical variation (inference on scatter) for each domain (size, regularity and density). During the data collection process, two groups of cells emerged in the molecular layer, based on the measured parameters, and two groups in the granular layer. Since it is well established that in the molecular layer, two types of interneurons exist, the basket cells and the stellate cells, we performed data analysis dividing the cells in these groups: cells with a mean length of the major axis of 11 µm (that we define stellate-like cells), and cells with a mean length of the major axis of 19.5 µm (that we define basket-like cells). Similarly, the granular layer contains at least two main groups: Golgi cells and granule cells, we hence labeled our two cell groups for analysis as (i) granule cells, with a major axis length up to 15 µm (most of the detected cells, with a very round and regular aspect); and (ii) Golgi-like cells, over 15 µm of major axis length (larger, more irregular cells).

### Multi-aspect testing and ranking inference

Separately for each type of cell (Basket = B, Stellate = S, Purkinje = P, Granules = Gr, Golgi = Go), the comparison of the morphometric descriptors (**Y**) among the three populations (M = male, F = female and FM = freemartin) has been formalized by the following statistical linear model:

1 $${\mathbf{Y}}_{ilj} = {{\varvec{\upmu}}} + {{\varvec{\uptau}}}_{lj} + {\varvec{\varepsilon}}_{ilj} ,$$

where specific location (**τ**_*lj*_) and scale effects **σ**^2^(**τ**_*lj*_) = **σ**^2^_*lj*_, *i* = B, S, P, Gr, Go, *j* = M, F, FM, are both allowed to differ across populations, while the random components ***ε ***are not specified in their distributional form according to a non-parametric permutation-oriented approach (Bonnini et al. 2014).

The inferential analysis to compare the sex-related groups has been formalized by the following null and alternative hypotheses:

2 $$\left\{ {\begin{array}{*{20}c} {H_{{0\left( {ljh} \right)}}^{{{\text{loc}}}} : \cap_{k} Y_{ljk} \mathop = \limits^{{{\text{loc}}}} Y_{lhk} \equiv \cap_{k} \left[ {\tau_{ljk } = \tau_{lhk } } \right]} \\ {\begin{array}{*{20}c} {H_{{1\left( {ljh} \right)}}^{{{\text{loc}}}} : \cup_{k} \left[ {\left( {Y_{ljk} \mathop < \limits^{{{\text{loc}}}} Y_{lhk} } \right) \cup \left( {Y_{ljk} \mathop > \limits^{{{\text{loc}}}} Y_{lhk} } \right)} \right]} \\ \end{array} } \\ { \equiv \cup_{k} \left[ {\left( {\tau_{ljk } < \tau_{lsk } } \right) \cup \left( {\tau_{ljk } > \tau_{lhk } } \right)} \right]} \\ \end{array} } \right.\quad \left\{ {\begin{array}{*{20}c} {H_{{0\left( {ljh} \right)}}^{{{\text{sca}}}} : \cap_{k} Y_{ljk} \mathop = \limits^{{{\text{sca}}}} Y_{lhk} \equiv \cap_{k} \left[ {\sigma_{ljk}^{2} = \sigma_{lhk}^{2} } \right]} \\ {\begin{array}{*{20}c} {H_{{1\left( {ljh} \right)}}^{{{\text{sca}}}} : \cup_{k} \left[ {\left( {Y_{ljk} \mathop < \limits^{{{\text{sca}}}} Y_{lhk} } \right) \cup \left( {Y_{ljk} \mathop > \limits^{{{\text{sca}}}} Y_{lhk} } \right)} \right]} \\ \end{array} } \\ { \equiv \cup_{k} \left[ {\left( {\sigma_{ljk}^{2} < \sigma_{lhk}^{2} } \right) \cup \left( {\sigma_{ljk}^{2} > \sigma_{lhk}^{2} } \right)} \right]} \\ \end{array} } \right.$$

where *l* = B, S, P, Gr, Go, *j,h* = M, F, FM, and *k* = 1,⋯,*p*, is the reference index for each univariate morphometric feature (see Table 2).

Permutation-based *p-*values (Corain and Salmaso 2015) have been calculated under the null hypothesis of approximated exchangeability. For details, see the supplemental materials.

To calculate the location and scatter ranking, respectively, we used the ranking methodology proposed by Arboretti et al. (2014).

### Computational issues

It is well known that resampling-based statistical methods, such as the permutation testing and ranking, we proposed in this paper, are quite demanding in computational power and time. In this view, there is a compelling need to optimize computational algorithms to make them more efficient and suitable for practical use. We implemented efficient permutation algorithms based on a previous R language version (Bonnini et al. 2014) under C language environment. The main difficulty was memory management because in a compiled language, there are no garbage collectors as in an interpreted language. By an in-depth simulation study, we proved that it was possible to considerably decrease the execution time even more when compiling the program with options of optimization. All codes and algorithms are freely available to all interested readers.

### Cluster K-means and principal component analysis (PCA)

Either for clustering and for visual representation purposes, two multivariate analysis techniques (i) cluster k-means; and (ii) Principal Component Analysis (PCA) analysis (Zelterman 2015) were applied on the whole dataset (i.e. on the three samples) to jointly handle all morphometric descriptors while considering the effects of all variables on the comparison between the three populations (male, freemartin and female).

### Statistical output guidelines

For each cerebellar cortical layer, statistical outputs were organized to highlight possible multivariate neural cell morphometric differences between the three sex-related populations. For each domain, we separately carried out:

1. Univariate analysis: for each morphometric descriptor we estimated the overall mean and the population-related shifts (parameters *μ* and *τ* as in model (1), paragraph multi-aspect testing and ranking inference.); underlying *p*-values were calculated via permutation symmetry testing approach (Bonnini et al. 2014). Since there are many cells per animal, the random sign-flip is performed block-wise within the cells of each animal. This is a way to account for subject-specific random effect (Finos and Basso 2014)
2. Multivariate testing and ranking analysis, where results were organized as two *p*-values 3 × 3 squared matrices of pairwise comparisons between groups. One matrix referred to the location and the other referred to the scatter analysis. Using all the cells above and below the diagonal, we represented in each squared matrix both the one-sided multivariate *p*-values to be associated to each one directional alternative (“greater than” and “lower than”). Finally, by exploiting the whole set of all relative *p-*value-based estimated dominances, we obtained a ranking suitable to sort the three populations from largest to the smallest (location ranking) and from most to least neuro-morphometric complex (scatter ranking).
3. Descriptive statistics: frequency distribution histograms representing the raw values of the first Principal Component Analysis (PCA) and a set of bivariate contour plots, showing the joint bivariate distribution. We kept the same set of colors across illustrations for the reference population (orange = female or F, green = Freemartin or FM, light blue = male or M). Concerning output interpretation, a population expected to be larger in location than another population should show a relative shift between the two related histograms (the smaller on the left and the larger on the right) and a right-up vs. left-down shift of the bivariate contour curves as well. Similarly, a population expected to be larger in scatter than another should present scattered and gathered histograms, respectively, and a wider vs. narrowed set of contour curves as well. From the inferential point of view, a larger location or scatter in a population over another will take a higher ranking and show a significant *p-*value in the associated row vs. column cell in the 3 × 3 squared matrix of pairwise comparisons.

## Results

### Gross anatomy of the bovine cerebellar vermis

**Fig. 1:**
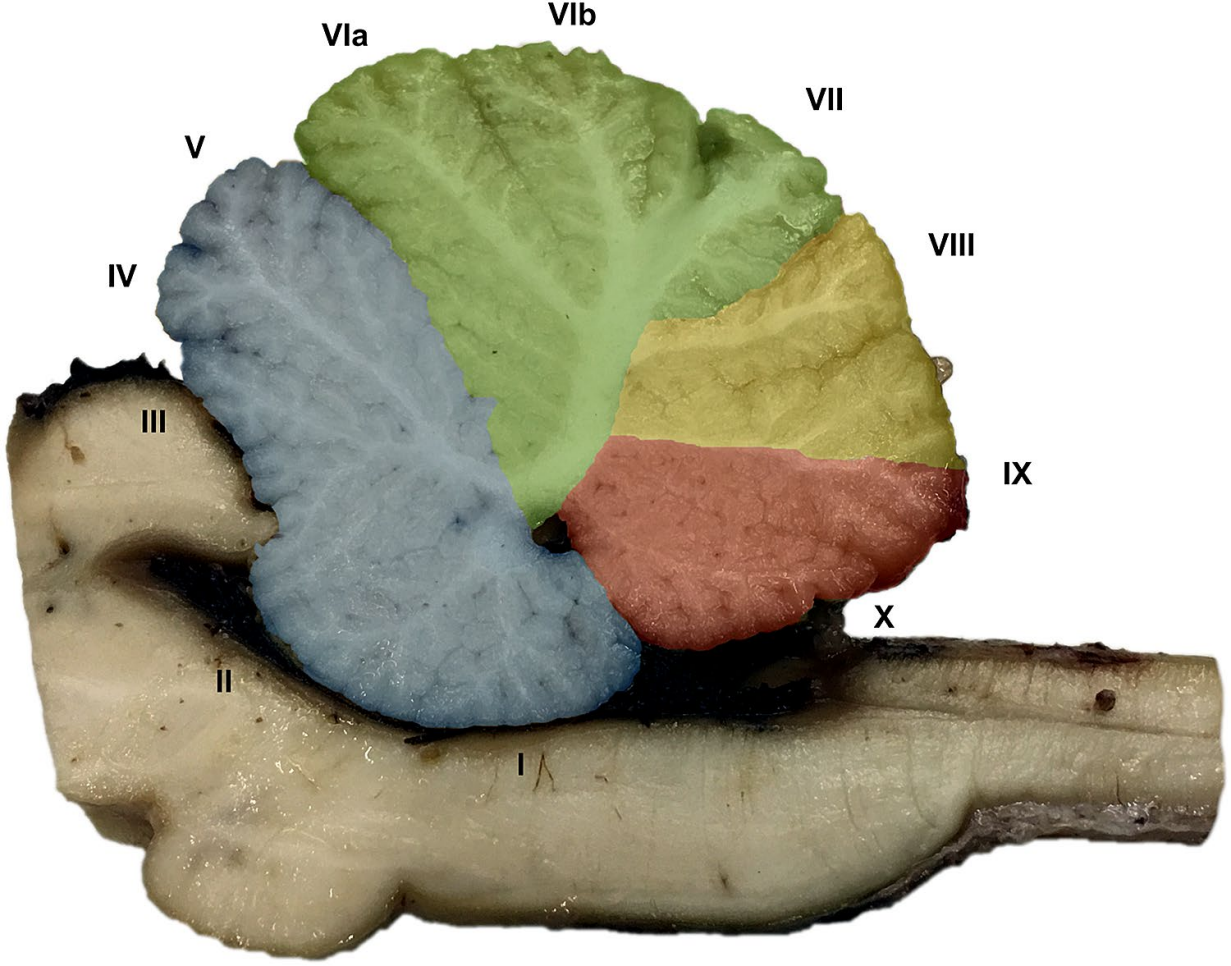
Sagittal section cut through the cerebellar vermis showing the foliation pattern divided along the anteroposterior axis into four transverse domains: anterior (blue; lobules I–V), central (green; lobules VI and VII), posterior (yellow; lobules VIII and anterior IX), and nodular (red; lobules posterior IX and X) (Ozol et al. 1999)

The adult bovine cerebellum is subdivided by an array of parasagittal and transverse fissures that yield a general irregular impression. On the whole, the major difference observed with the well-known anatomy of the primate cerebellum is the prevalence of the vermis over the lateral lobes, a feature typical of large herbivore mammals like Perissodactyls and terrestrial (but not marine) Cetartiodactyls (Fig. 1). However, notwithstanding the differences with the human cerebellum, here we adopted for the vermis the internationally recognized nomenclature of the folia (Larsell 1952, 1953; Larsell and Jansen 1970). The specific organization of the bovine cerebellum has been described by Barone and Bortolami (2004). The morphology of the vermis varied among the subjects of our experimental series, but the general plan was always recognizable.

### Histology of the cortex in the lobules VIII-IX of the bovine vermis

**Fig. 2:**
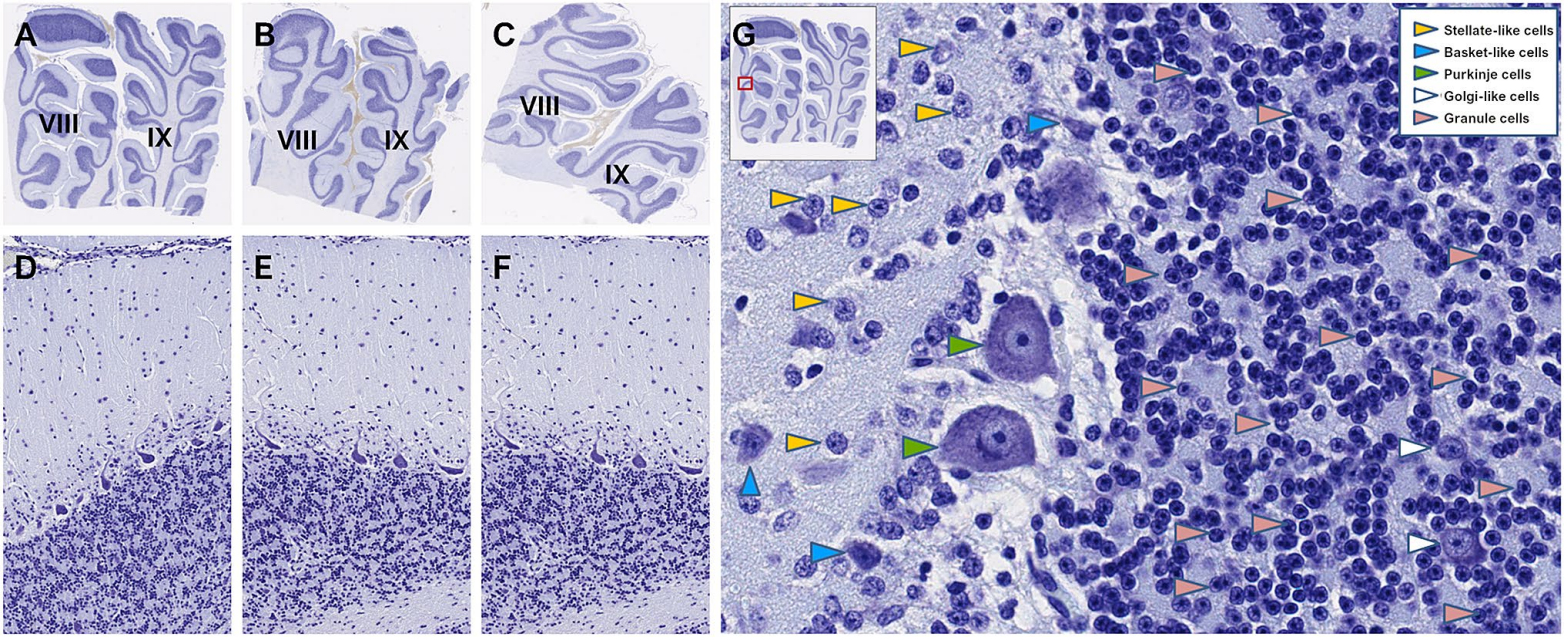
Photomicrographs of 8-µm-thick parasagittal Nissl-stained sections showing the topographical organization of the bovine lobule VIII and lobule IX. Male, female and freemartin (**a**, **b** & **c** ×1; **d**, **e** & **f** ×20) showing the three-layered organization. Purkinje cells are located in a monolayer between the inner granular layer and the outer molecular layer of the cortex. Panel **g**: (×200) photomicrograph showing the five kinds of neural cells characterized in the different layers. The red arrows indicate the basket-like cells, green arrows indicate the stellate-like cells, yellow arrows indicate the Purkinje cells, blue arrows indicate the granules and white arrows indicate the Golgi-like cells

Histology of the cerebellar cortex was uniform over the entire lobules VIII-IX of the sections of the vermis that we analyzed (Fig. 2a–c). The classic three layers were easily identified (Fig. 2d–f). The outer molecular layer appeared relatively thick, containing few sparse cells immersed in the glia (Fig. 2d–f). The characteristic basket and stellate neurons were easily recognized (Fig. 2g). The Purkinje monolayer contained the somata of Purkinje neurons aligned between the molecular and the granular layer (Fig. 2g). The granular layer presented the usual densely-packed population of small, round granules. Few larger Golgi type II neurons were also seen (Fig. 2g).

### Morphometric data analytics results

#### External molecular layer

##### Stellate-like cells

**Fig. 3:**
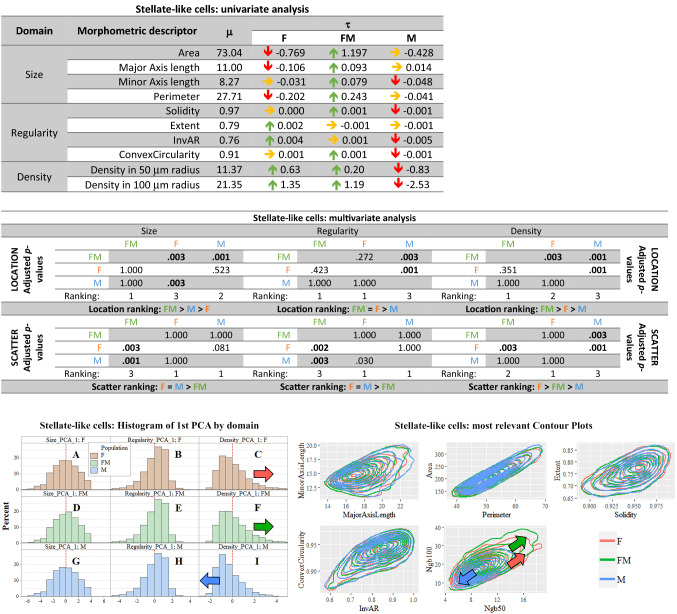
**Stellate-like summary. In red female population (F), in blue male population (M), in green freemartin population (FM). First table:** Results of univariate analysis performed in the stellate-like cells of the molecular layer. For each morphometric descriptor, estimates of parameters *m* (overall mean across the three populations), *t* (specific mean difference for each population). The green arrow means a 1% significantly greater than zero estimate, the red arrow means a 1% significantly lower than zero estimate. Underlying* p*-values were calculated via permutation symmetry testing approach. The yellow arrow indicates no significant difference from zero. We are referring to raw* p*-values, i.e. without any kind of adjustment by multiplicity. **Second table:** Multivariate analysis by domain and aspect for stellate-like cells. Between-populations location and scatter one-sided adjusted permutation* p*-values are presented in squared matrices. In each cell, the alternative hypothesis is “population-in-row is larger than population-in-column”. The 5% significant* p*-values are highlighted in bold. Location and scatter rankings are derived from dominance in pairwise comparisons. **Left figure:** Histograms representing the raw values of the first principal component analysis (PCA) of stellate-like cells in the molecular layer, by domain (size, regularity and density) and population (female, male and freemartin). This descriptive method helps visualizing the multidimensional comparisons across population. Left or right shifting shows that the related population likely takes lower or larger value for the given domain. Scatter translates into how gathered or spread data are. Coloured arrows highlight the shift directions. **Right figure:** Contour plots representing the bivariate joint distribution of the descriptors (in pairs) for the stellate-like cells. A relative down-left or top-right shift shows the related population likely takes lower or larger value in that domain. Spread of the contour translates scatter of the data. Coloured arrows highlight the large observed shifts among populations. The red and green arrows indicate that female and freemartin possess a larger and more scattered cell density

*Univariate analysis in the stellate-like cells* The body of the stellate-like cells had an average area of 73 μm^2^, an average perimeter of 28 μm and major and minor axes average lengths of 11 and 8 μm, respectively (see Fig. 3, first table). Considering the size domain, the perimeter and major axis length showed that female stellate-like cells were significantly smaller than in male as indicated in the *τ* column (red arrows). Conversely, the minor axis lengths of stellate-like cells were significantly inferior in males (red arrows). In freemartins, stellate-like neurons were larger in size than both in females and males (green arrows). Regarding the regularity domain, the stellate-like cells in both females and freemartins showed significantly more regular soma than in males. For the density domain, stellate-like cells in male were less densely packed than in females and freemartins.

*Multivariate analysis and principal component analysis (PCA) of stellate-like cells* The multivariate inferential location and scatter analysis are summarized in the Fig. 3-second table. Regarding the size domain, the stellate-like cells in females were significantly smaller (*p-*value 0.003), more regular (*p*-value 0.001) and denser (*p*-value 0.001) than in males. In freemartins, stellate-like cells were larger in size than both in females and in males (*p*-value 0.003 and 0.001), and denser than both males and females (both *p*-values = 0.003).

The multivariate inferential scatter analysis showed that female stellate cells had a significantly more scattered distribution than in males density-wise (*p*-value 0.001), while freemartins had the most homogeneous distribution than both males and females in size (*p*-values 0.003 and 0.001) and regularity (*p*-values 0.002 and 0.003). Conversely, freemartins’ density indicators were intermediate between males (*p*-values 0.003) and females (*p*-value 0.003), as shown in Fig. 3, second table.

The graphical representation by PCA showed no difference in distribution, except for density between males *vs*. females and freemartins (Fig. 3, left figure c, f and i). A graphical representation by contour plots suggested differences only in density (Fig. 3, right figure), in line with the PCA analysis.

##### Basket-like cells

**Fig. 4:**
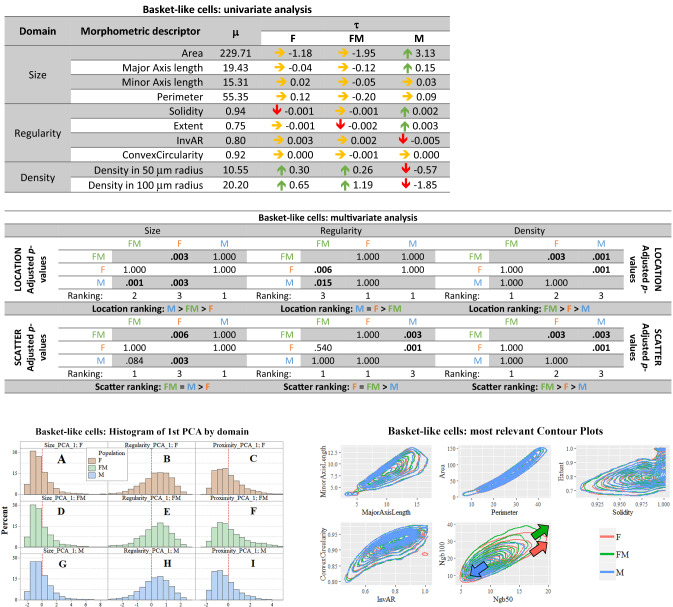
**Basket-like cells summary. In red female population (F), in blue male population (M), in green freemartin population (FM). First table:** Results of univariate analysis performed in the basket-like cells of the molecular layer. For each morphometric descriptor, estimates of parameters* m* (overall mean across the three populations),* t* (specific mean difference for each population). The green arrow means a 1% significantly greater than zero estimate, the red arrow means a 1% significantly lower than zero estimate. Underlying* p*-values were calculated via permutation symmetry testing approach. The yellow arrow indicates no significant difference from zero. We are referring to raw* p*-values, i.e. without any kind of adjustment by multiplicity. **Second table:** Multivariate analysis by domain and aspect for basket-like cells. Between-populations location and scatter one-sided adjusted permutation* p*-values are presented in squared matrices. In each cell, the alternative hypothesis is “population-in-row is larger than population-in-column”. The 5% significant* p*-values are highlighted in bold. Location and scatter rankings are derived from dominance in pairwise comparisons. **Left figure:** Histograms representing the raw values of the first principal component analysis (PCA) of basket-like cells in the molecular layer, by domain (size, regularity and density) and population (female, male and freemartin). This descriptive method helps visualizing the multidimensional comparisons across population. Left or right shifting shows that the related population likely takes lower or larger value for the given domain. Scatter translates into how gathered or spread data are. Coloured arrows highlight the shift directions. **Right figure:** Contour plots representing the bivariate joint distribution of the descriptors (in pairs) for the basket-like cells. A relative down-left or top-right shift shows the related population likely takes lower or larger value in that domain. Spread of the contour translates scatter of the data. Coloured arrows highlight the large observed shifts among populations. The red and green arrows indicate that female and freemartin possess a larger and more scattered cells density

*Univariate analysis in the basket-like cells* Basket-like cells had an average area of 230 μm^2^, and an average perimeter of 55 μm. Their major and minor axes lengths were averaged 19 μm and 15 μm, respectively (see Fig. 4, first table). Regarding size, male basket-like cells appeared significantly larger in area and major axis than in females and in freemartins. In the regularity domain, both female and freemartin basket-like cells seemed less regular than male ones. Regarding density, male basket-like cells were much more isolated than female and freemartin cells.

*Multivariate analysis and PCA of basket-like cells* The multivariate inferential location and scatter analysis is summarized in the Fig. 4, second table. Female basket-like cells were significantly smaller (*p* = 0.003) and denser (*p* = 0.001) than in males. Freemartin basket-like cells were larger than in females (*p* = 0.003) and smaller than in males (*p* = 0.001), while they were less regular than both in males (*p* = 0.015) and females (*p* = 0.006). Freemartin basket-like cells were also the densest group (*p* = 0.003 Ngb 50, *p* = 0.001 Ngb 100) compared to males and females (see Fig. 4, second table).

The multivariate inferential scatter analysis showed that basket-like cells in females had a significantly less scattered distribution than in males (*p* = 0.003) and a more scattered distribution for both regularity (*p* = 0.001) and density (*p* = 0.001). In freemartins, basket-like cells had a more heterogeneous distribution in size than in females (*p* = 0.006) and in regularity than males (*p* = 0.003). Regarding the density indicators, freemartins had a more scattered distribution than both females (*p* = 0.003) and males (*p* = 0.001), as shown in Fig. 4, second table.

The graphical representation by PCA did not show any large shift among sexes in any domain (Fig. 3, right figure). However, contour plots for the domain density transcribed the inferential location and scatter analysis results. A difference could be found in density, where female basket-like cells were denser than in males.

#### The Purkinje layer

##### Univariate analysis in the Purkinje cells

Results confirmed that Purkinje neurons are the largest cerebellar cell type. Purkinje neurons had an average area of 2500 μm^2^, an average perimeter of 250 μm and major and minor axis average lengths of 70 μm and 50 μm, respectively (Fig. 6, first table). Female Purkinje cells appeared significantly larger in area, major axis length and perimeter. Male Purkinje cells had a significantly smaller perimeter, were more regular in shape, but more sparsely distributed. In freemartins, Purkinje neurons showed an intermediate size and density between female and male. The three sex groups had no significant difference in scatter.

#### Multivariate analysis and PCA of the Purkinje cells

**Fig. 5:**
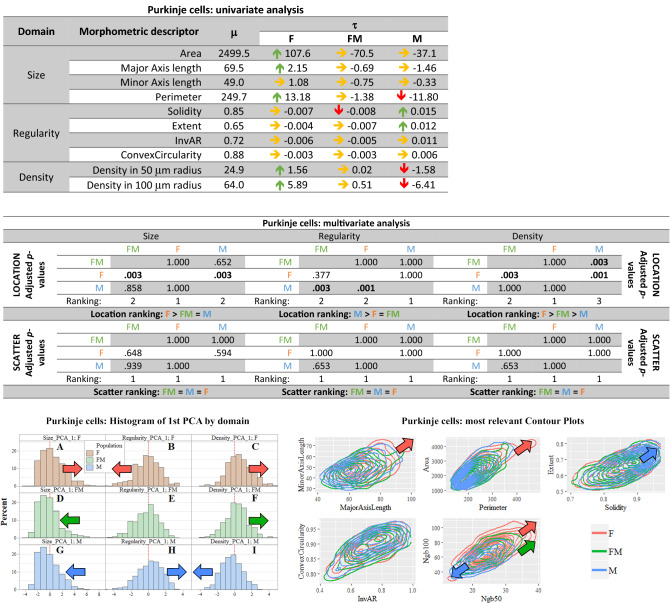
**Purkinje cells summary. In red female population (F), in blue male population (M), in green freemartin population (FM). First table:** Results of univariate analysis performed in the Purkinje cells of the molecular layer. For each morphometric descriptor, estimates of parameters* m* (overall mean across the three populations),* t* (specific mean difference for each population). The green arrow means a 1% significantly greater than zero estimate, the red arrow means a 1% significantly lower than zero estimate. Underlying* p*-values were calculated via permutation symmetry testing approach. The yellow arrow indicates no significant difference from zero. We are referring to raw* p*-values, i.e. without any kind of adjustment by multiplicity. **Second table:** Between-populations (female, male and freemartin) multivariate analysis by domain and aspect. Between populations location and scatter one-sided adjusted permutation* p*-values are presented in squared matrices where in each cell the alternative hypothesis refers to “population-in-row is larger than population-in-column”. The 5% significant* p*-values are highlighted in bold. By exploiting dominance results from pairwise comparisons, location and scatter ranking are finally derived. **Left figure:** Histograms representing the raw values of the first principal component analysis (PCA) of Purkinje cells, by domain (size, regularity and density) and population (female, male and freemartin). This descriptive method helps visualizing the multidimensional comparisons across population. Left or right shifting shows that the related population likely takes lower or larger value for the given domain. Scatter translates into how gathered or spread data are. Coloured arrows highlight the shift directions. **Right figure:** Contour plots representing the bivariate joint distribution of the most relevant descriptors (considered in pairs) for the Purkinje cells. As the contours show a relative down-left or top-right shift, the related population is likely to take lower or larger value on that domain. More gathered or spread contour profiles indicate relatively smaller or larger scatter. The coloured arrows are set up or order to highlight the larger observed shifts

The non-parametric inferential multivariate testing and ranking results for the location and scatter of Purkinje cells are summarized in Fig. 5, second table.

Purkinje cells in females were found to be significantly larger (*p* = 0.003), more irregular (*p* = 0.001) and denser (*p* = 0,003) than in males. Freemartin Purkinje cells showed an intermediate pattern between females and males. They were smaller in size than in females (*p*-value 0.003), and also less regular (*p*-value 0.003) and denser (*p*-values 0.003) than in males, as shown by PCA (Fig. 5, left figure). The PCA also showed shifts in the distributions. Specifically, female Purkinje neurons took more often higher size values (right shift) than in both males and freemartins. Female Purkinje neurons’ larger shape irregularity can be seen in the left shift of the regularity domain. Female and freemartin Purkinje cell density also displayed higher values more frequently, as shown in the PCA relative to density. No significant difference was found with the scatter analysis.

Contour plotting revealed the larger size and higher density of female Purkinje cells, and the relative lower density of male neurons (Fig. 5, right figure).

#### Granular layer

##### Granule cells

**Fig. 6:**
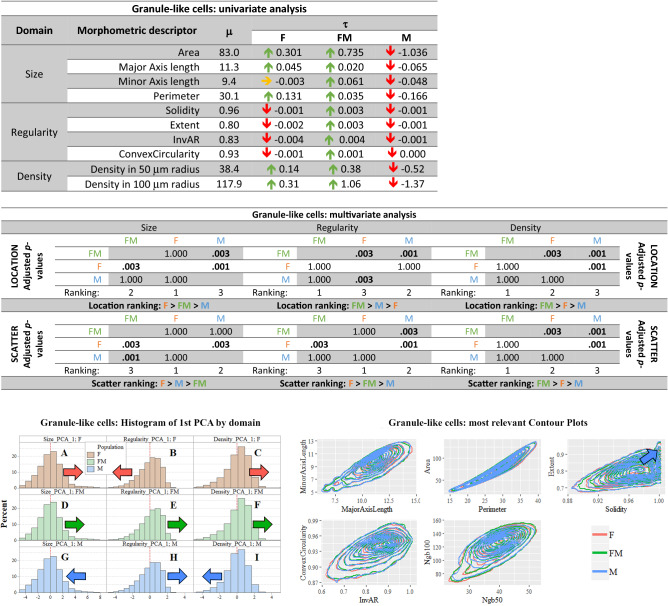
**Granule cells summary. In red female population (F), in blue male population (M), in green freemartin population (FM). First table:** Results of univariate analysis performed in the granule cells of the molecular layer. For each morphometric descriptor, estimates of parameters* m* (overall mean across the three populations),* t* (specific mean difference for each population). The green arrow means a 1% significantly greater than zero estimate, the red arrow means a 1% significantly lower than zero estimate. Underlying* p*-values were calculated via permutation symmetry testing approach. The yellow arrow indicates no significant difference from zero. We are referring to raw* p*-values, i.e. without any kind of adjustment by multiplicity. **Second table:** Multivariate analysis by domain and aspect for granule cells. Between-populations location and scatter one-sided adjusted permutation* p*-values are presented in squared matrices. In each cell, the alternative hypothesis is “population-in-row is larger than population-in-column”. The 5% significant* p*-values are highlighted in bold. Location and scatter rankings are derived from dominance in pairwise comparisons. **Left figure:** Histograms representing the raw values of the first principal component analysis (PCA) by domain (size, regularity and density) and population (female, male and freemartin) of Golgi-like cells. This descriptive method helps visualizing the multidimensional comparisons across population. Left or right shifting shows that the related population likely takes lower or larger value for the given domain. Scatter translates into how gathered or spread data are. Coloured arrows highlight the shift directions. **Right figure:** Contour plots representing the bivariate joint distribution of the most relevant descriptors (considered in pairs) for the granule cells. Since the graphical representation showed no observable differences, no arrow has been indicated

*Univariate analysis in the granules* Granules are the smallest cerebellar neurons. Their soma had an average area of 83 μm^2^, and an average perimeter of 30 μm. The major and minor axes lengths were 11 μm and 9 μm, respectively (Fig. 6, first table). The male granules appeared significantly smaller in all size, regularity and density descriptors. Freemartin granules showed values similar to females in size and higher in density, but also the highest regularity (Fig. 6, first table).

*Multivariate analysis and PCA of granules* The multivariate inferential location and scatter analysis are summarized in the Fig. 6, second table.

Female granules were larger (location aspect, *p* = 0.003), more irregular in shape (*p* = 0.003) and denser (*p* = 0.003) than in males, but their values also varied more, both in size and density (*p*-values 0.003 and 0.001). Freemartin granules showed an intermediate size, and were the most regular and densest population. Freemartin granules had the most homogeneous population for size (*p*-values 0.003 and 0.001), and more homogeneous than males in regularity (*p*-values 0.003). However, the freemartin granules had the most heterogeneous distribution for density indicators (*p*-values 0.003 and 0.001). Representation of PCA illustrated the location results. Although the distributions seem similar, small shifts can be seen. Male distribution shifted to the left in size and density, while females and freemartins moved to the right. The regularity shifts were opposite, except for freemartins (Fig. 6, left figure).

The multivariate inferential scatter analysis showed that female granules showed a significantly more scattered distribution than male granules both in size and density (*p*-values 0.003 and 0.001). Contour plots did not translate the inferential location and scatter analysis differences found (Fig. 6, right figure).

##### Golgi-like cells

**Fig. 7:**
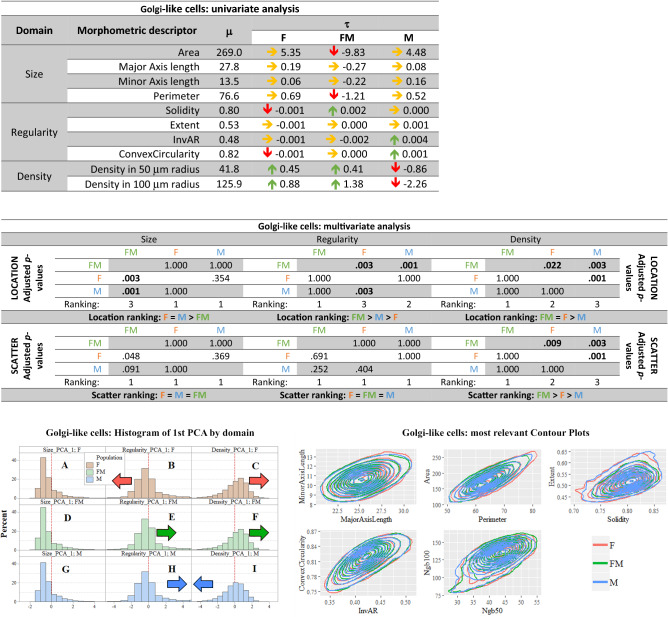
**Golgi-like cells summary. In red female population (F), in blue male population (M), in green freemartin population (FM). First table:** Results of univariate analysis performed in the Golgi-like cells of the molecular layer. For each morphometric descriptor, estimates of parameters* m* (overall mean across the three populations),* t* (specific mean difference for each population). The green arrow means a 1% significantly greater than zero estimate, the red arrow means a 1% significantly lower than zero estimate. Underlying* p*-values were calculated via permutation symmetry testing approach. The yellow arrow indicates no significant difference from zero. We are referring to raw* p*-values, i.e. without any kind of adjustment by multiplicity. **Second table:** Multivariate analysis by domain and aspect for Golgi-like cells. Between-populations location and scatter one-sided adjusted permutation* p*-values are presented in squared matrices. In each cell, the alternative hypothesis is “population-in-row is larger than population-in-column”. The 5% significant* p*-values are highlighted in bold. Location and scatter rankings are derived from dominance in pairwise comparisons. **Left figure:** Histograms representing the raw values of the first principal component analysis (PCA) by domain (size, regularity and density) and population (female, male and freemartin) of Golgi-like cells. This descriptive method helps visualizing the multidimensional comparisons across population. Left or right shifting shows that the related population likely takes lower or larger value for the given domain. Scatter translates into how gathered or spread data are. Coloured arrows highlight the shift directions. **Right figure:** Contour plots representing the bivariate joint distribution of the most relevant descriptors pairs in Golgi-like cells. As the contours show a relative down-left or top-right shift, the related population is likely to take lower or larger value on that domain. More gathered or spread contour profiles are also indication of possible relatively smaller or larger scatter parameters. The coloured arrows are set up or order to highlight the larger observed shifts

*Univariate analysis in the golgi-like cells* Golgi type II neurons in the granular layer had an average area of 269 μm^2^, an average perimeter of 77 μm, with major and minor axes length average values of 28 μm and 13.5 μm, respectively (Fig. 7, first table). Only a few differences were observed across sex groups. Freemartin Golgi type II neurons were significantly smaller in area and perimeter. Male and freemartin Golgi type II neurons were both more regular in shape than in females, whereas females and freemartins were more densely aggregated than males.

*Multivariate analysis and PCA of golgi type II neurons* The multivariate inferential location and scatter analysis are summarized in Fig. 7, second table. Golgi type II neurons presented no difference in the size domain between male and female (*p*-value 0.354). Significant differences were found in shape regularity and density, with female Golgi type II neurons being more irregular (*p*-value 0.003) and closer together (*p*-value 0.001) than in males. This can be seen in the PCA in the female and male shifts in distributions signaled by arrows (Fig. 7, left figure). In freemartins, Golgi type II neurons showed smaller size than both females (*p*-value 0.003) and males (*p*-value 0.001). They were also the most regular (*p*-values 0.003 for females and 0.001 for males) and the densest group (*p*-values 0.022 and 0.003). Multivariate inferential scatter analysis showed that female Golgi type II neurons had a significantly more variability in values than males only in the density domain (*p*-value 0.003). Freemartins, on the other hand, had the most heterogeneous distribution for density domain (*p*-values 0.009 and 0.003 for female and male, respectively, Fig. 7, second table). Graphical representation by contour plots did not show observable differences (Fig. 7, right figure).

### Final remarks on ranking analyses

A summary of all ranking analyses is shown in Table 3. Purkinje cells and the granules show the same pattern of results in male *vs.* female comparison. The female population displays larger, irregular cell bodies more closely arranged than in males. This setting also holds for Golgi type II neurons. Freemartin Purkinje cells are similar to those of males in size and density, but show less regularity in the shape of their soma. Freemartin granules have an intermediate position between females and males for their size, but had the roundest, more closely arranged together cell bodies, which holds true also for the Golgi type II neurons. Finally, it is worth noting that the male cerebellum displayed the lowest cell density for all the five cell types analyzed. Conversely, freemartins showed the densest pattern across cell types, except for Purkinje neurons, which were most closely laid in females (Table 3).

**Table 3:** Summary of ranking results by type of cell and domain

Ranking	Size	Regularity	Density
Stellate-like cells	Location: FM > M > F Scatter: F = M > FM	Location: FM = F > M Scatter: F = M > FM	Location: FM > F > M Scatter: F > FM > M
Basket like cells	Location: M > FM > F Scatter: M = FM > F	Location: M = F > FM Scatter: FM = F > M	Location: FM > F > M Scatter: F > FM > M
Purkinje cells	Location: F > FM = M Scatter: F = FM = M	Location: M > F = FM Scatter: FM = F = M	Location: F > FM = M Scatter: F = FM = M
Granule cells	Location: F > FM > M Scatter: F > M > FM	Location: FM > M > F Scatter: F > FM > M	Location: FM > F > M Scatter: FM > F > M
Golgi-like cells	Location: F = M > FM Scatter: F = FM = M	Location: FM > M > F Scatter: F = FM = M	Location: FM > F > M Scatter: FM > F > M

## Discussion

The goal of this study was to quantify cytoarchitectonic differences in the lobules VIII and IX of the vermis of the bovine cerebellum. Morphometric data collected from neural cells have been analyzed by a new alternative statistical approach based on multi-aspect testing and a ranking method. The choice of *Bos taurus* as an experimental model allowed us a comparison among males, females and the naturally occurring intersex freemartins.

This work was designed to answer two basic questions: (1) does the cerebellar cortex show morphometric differences in the cytoarchitecture among male, female and freemartins? and (2) which aspect of each cerebellar layer presents sexual differences? The output of this multi-aspect approach produced a detailed overview of the neuronal morphology of lobules IIIV and IX examined in males, females and intersex individuals.

### Why dimorphism in lobules VIII and IX of the vermis?

The cortex of the mammalian cerebellum shows a rather constant organization throughout the different lobules, but different regions control diverse functions (Glickstein et al. 2009). Recent studies suggest the presence of structurally (Fan et al. 2010) and physiologically (Abel et al. 2011) dimorphic areas in the human cerebellum. Fan and colleagues (2010) concluded that males exhibit higher leftward asymmetry within a few lobules and lower rightward asymmetry mainly within the Crus II lobule, compared to females. Additionally, MRI imaging determined that frontal and medial posterior cerebellar lobes were larger in males, while the female lateral posterior lobe presented larger volumes of gray matter. Since precise characterization of gender-related differences in the cerebellar cortex remains inconclusive, anatomically unspecific or even conflicting, we decided to focus our attention on structural gender differences in the paleocerebellar lobules IIIV and IX, at the caudal base of the vermis.

### Purkinje neurons and granules show the same morphological pattern in males and females

Since Purkinje neurons form the output of the cerebellum, most of the studies related to gender morphological differences in the cerebellar cortex concern Purkinje neurons. Males have been found to have more numerous and larger Purkinje neurons than females (Wittmann and McLennan 2011). In a pioneering morphological study in the human cerebellum, Hall and colleagues found that the number of Purkinje cells was similar between sexes across ages, and that the difference in numbers could be linked to absolute difference in size between male and female cerebelli (Hall et al. 1975). Notwithstanding absolute number and size differences, our results show that in the bovine female, Purkinje neurons were larger, more irregular and denser than in males, while Purkinje cells in freemartins had an intermediate position.

Multi-aspect analysis demonstrated that male and female Purkinje neurons and granules show a common morphological pattern. However, female Purkinje neurons and granules are larger, more irregular and denser than in males. This result was confirmed by the location ranking inference (Table 2). To the best of our knowledge, there are no specific studies to compare our data with, and thus support or contradict our results. It is, however, interesting to note that earlier work from our laboratory in which we analyzed the capacity of estradiol (E2) to affect the growth of male and female bovine cerebellar granules in culture (Montelli et al. 2017), showed that female granules always showed significantly larger morphological values than the corresponding category of male cells. Additionally, male granules treated with E2 became similar in size to female controls. Moreover, the E2 treatment exerted a stronger trophic effect in female than in male granules. The trophic effects exerted from the higher natural concentration of E2 in females could explain the larger size of female Purkinje neurons and granules. These effects could be differential across cell types, and to improve our understanding of sex-related differences, and finely characterize them, gender should be related to the precise regional morphology of the cerebral or cerebellar tissue.

### The importance of structural differences between genders in the cytoarchitecture of the cerebellum

We must consider that the incidence and severity of some neurodegenerative diseases and neuropsychological conditions differ significantly in the two sexes (Swaab 2003). Examples include Alzheimer's disease (higher prevalence in women), Parkinson's (higher prevalence in men) and autism (higher incidence in boys) (Alzheimer's Association 2014, Hanamsagar and Bilbo 2016; Van Wijngaarden-Cremers et al. 2014). The study of sex differences in the brain might contribute to a better understanding of these conditions (Abel et al. 2011). However, the number of published studies using a male-only cohort is strikingly high, and studies on females need to be increased (Zucker and Beery 2010).

The organization and structure of the lobules are relatively constant in mammals, thus suggesting that their ontogeny is controlled by morphogenetic programs (Sudarov and Joyner 2007). Therefore, pathology can affect these programs, as in autistic patients, which show significantly smaller lobules VI and VII of the vermis (Courchesne et al. 1988) and an increased cerebellar volume compared to controls using MRI imaging (Sparks et al. 2002). Similarly, patients suffering from bipolar disorders show higher volumes of the male vermis (Womer et al. 2016).

The study of the differences in constituents of the brain circuitry represents a first step to gain a better understanding of the pathophysiology of a given disease. Arguably, sex differences in the morphology of neuronal populations may influence connecting patterns and hence function. Therefore, a fine analysis and characterization of cytoarchitecture can help appreciate overall function and pathology. Image analysis of histological sections routinely provides valuable data on the morphology of multiple cells, acquired and analyzed individually. However, comparison of different populations defined by factors, such as sex, age or pathological condition, requires suitable multivariate inferential methods (Maney 2016) to yield meaningful and trustable data.

### A multi-aspect testing solution for a multivariate problem

In biosciences, standard testing is usually performed by univariate statistical analyses based on the means (location) of any single morphometric descriptor. These methods carry two main drawbacks: (1) they do not consider the variability (scatter) aspect of the data; and (2) they do not jointly consider different parameters and, therefore, they do not allow more than limited outlooks on the results (Roseblatt 2016).

To quantify the cytoarchitectural differences among male, female and intersex populations, we applied a novel non-parametric inferential multi-aspect testing and ranking approach, conceived as an extension of the permutation and combination-based testing methodology (Bonnini et al. 2014; Corain and Salmaso 2015; Corain et al. 2016). The main advantage of this proposed multivariate inferential approach is the possibility (1) to analyze data separately for each distributional aspect, i.e. mean (inference on location), and variance (inference on scatter) see (Peruffo et al. 2019); and (2) to analyze data specifically for each domain (size, regularity and density), to test directionally the hypothesis equality *vs*. dominance for the three sex-categories of the bovine populations that we describe here. The result is an array of multiple parameters’ variance and mean that can be plotted against each other and give a better view of interactions, and possibly help identify determinant relationships and characteristics for each population.

### Multi-aspect testing in neuroanatomy

Morphometry, location and scatter aspects can be associated to two separate issues: sexual dimorphism-related neurotrophic factors (Carrer and Cambiasso 2002) and neural complexity (Johnson et al. 2008). Inferring on location, it is possible to state if there is evidence for neuronal dominance in size or in density, while considering cell regularity as well. Inferring on scatter, one can state if there is evidence for a cytoarchitecture pattern. If by cytoarchitecture complexity we refer to a measure of heterogeneity of neural cells’ morphological features in a region of interest, a more complex cytoarchitecture will show a larger scatter distribution of the cells’ morphological features, while a less complex cytoarchitecture will show a smaller scatter distribution in morphological features.

### Computational issues underlying the multi-aspect testing

Permutation-based multivariate and multi-aspect testing and ranking were performed by ad hoc scripts written in R package, available upon request. It is well known that resampling-based statistical methods, such as permutation tests, are quite demanding in computational time and power. Efforts are now being made to test and optimize computational algorithms. One of the possible ways is the use of computational languages, and studies have been made to discuss comparisons of execution times with parallel implementations in R versus C languages (Carvajal-Schiaffino et al. 2016). Parallel computing is a programming technique where instructions can be executed simultaneously by different processor cores. This technique is based on dichotomy, the principle that big problems can be divided into smaller parts that can be solved concurrently. The abstraction level of the R language that makes it user-friendly necessitates an interpreter and does not permit an optimal use of the memory and cores allowed in the parallel version. Additionally, parallel scripting in R cannot use all processor cores efficiently. Therefore, a compiled version of the algorithm is then faster than the interpreted version.

### Nissl staining: the stain showing the cytoarchitecture organization in the brain

Nissl staining is still the most widely accepted stain showing the regional or laminar organization of the cytoarchitecture in the different brain areas (Pilati et al. 2008). Throughout this study, thionine-based Nissl staining confirmed to be an easy and efficient method to identify single neurons and the overall neuronal cytoarchitecture. It is adapted to careful quantitative studies where entire populations of cells must be assessed (García-Cabezas et al. 2016), since Nissl technique stains the entire population of cells in nervous tissue and can be used to label neurons and glial cell types in stereological counts in the brain. Other advantages of Nissl staining over immunohistochemistry are its low cost and the abundant available material from different species already processed in neuroscience laboratories worldwide, thus including human archival samples (García-Cabezas et al. 2016). In the perspective of world-wide cloud-based automatization algorithms, standardized stains would help confront results. Necessarily, there are limitations to Nissl staining and, independently, to automatic processing. Aside from technical shortcomings, such as poor fixation, low quality sampling, and maladapted processing, at each step for fragile neural tissue, the best Nissl stain will color, without discrimination, glial and neuronal somata only. Depending on the thickness of the section, some large cells can be incomplete, and therefore yield inaccurate results of their morphology, a potential bias to which stereology has attempted to respond. However, in a cohort of a very large number of cells, the mean cell morphologies should emerge appropriately.

There have been numerous studies using cell shape in some form to assess neuronal populations in the brain (Buxhoeveden et al. 2001; Amunts et al. 2007a; Semendeferi et al. 2011; Spocter et al. 2018), as noted by Spocter and colleagues (2018) in a report on Cetartiodactyls, methods vary even among similar types of study, and comparison is seldom possible. The study of the neuropil according to Spocter et al. (2018), is a useful proxy to study connectivity in the isocortex, but by design, the measure of intercellular space (including microvasculature and dendrites) applied on a binary thresholded 2D image cannot measure the neuropil spaces within the depth of the 50 µm of the stained section. The technique in itself can provide information on the number of cells (alternatively neuropil) in a neural area, but does not generate data on cell morphology. Another method is the gray level index (GLI) used by Amunts et al. (2007a, b), which also starts from a gray image and determines a cell quantity profile along an axis perpendicular to the pial surface, where black is expected to depict cell bodies and light gray/white is the intercellular space. This method suits perfectly the detection of variations in cortical structure to identify transition areas (Amunts et al. 2007a), but does not account for basic cell morphometry, or subtle changes in layers. Instead, very specific studies have focused on substructures in the cortex (Buxhoeveden et al. 2001; Semendeferi et al. 2011). These specifically designed studies do use Nissl-stained material, digitalized and analyzed by means of indirect morphometric descriptors including column width and gray level ratio to account for cell density or neuropil space. While very adapted to their specific goal and robust in their output, the focus, and therefore the unit, in these latter studies remains the substructure and not the cell, which is what we strived for in the present study.

## Conclusion

Unraveling the structural and functional complexity of specific brain regions remains a hard task. This is true especially in the field of neurodegenerative diseases that are sexually dimorphic, in which case one sex is usually much less subjected to the disease than the other. New methodologies that promote interdisciplinary collaboration are required to overcome potential obstacles (DeFelipe 2015) and understand the underlying protection/prevalence processes.

Neuroanatomical research on the sex differences in the central nervous system is still currently a relatively controversial field, and in this sense, a goal should be to take into consideration male and female models (Zucker and Beery 2010; Montelli et al. 2017), to separate data by sex and when possible, compare the sexes (Maney 2016). The performance of multivariate and multi-aspect statistical methods could provide a robust base for tissue screening and finer analysis overcoming limitations of traditional testing, such as the ability to include more variables.

The method can be easily applied especially in the field of neurodegenerative pathologies where structural differences represent the anatomical substrate underlying the functional differences between the two sexes. The present cerebellar study advances knowledge in the field of sex-related cerebellar dimorphisms and emphasizes that the bovine freemartin syndrome represents a stimulating model to explore gender differences in translational neuroscience.

## Electronic supplementary material

Below is the link to the electronic supplementary material.

Supplementary file1 (DOCX 24 kb)

## Data Availability

The code can be freely given upon request.
