# Supplementary material for: Multi-aspect testing and ranking inference to quantify dimorphism in the cytoarchitecture of cerebellum of male, female and intersex individuals: a model applied to bovine brains
Source: Brain Struct Funct. 2020 Sep 28;225(9):2669–88. doi: 10.1007/s00429-020-02147-x (PMC7674367; doi:10.1007/s00429-020-02147-x)
Supplement: Supplementary file 1 — Supplementary file1 (DOCX 24 kb) [file 429_2020_2147_MOESM1_ESM.docx]

Supplemental materials - *Statistical modelling and data analytics*

The following supplementary material covers *in extenso* the statistical methods used in the present work. Raw data on the morphometrics of our cerebellum samples can be requested directly to the authors, although it is unnecessary for the clarity of the theory.

In order to formalize the comparison among the three populations under investigation, i.e. M=male, F=female and FM=freemartin, we assume an ANOVA-type two-way experimental data representation model. More formally and without loss of generality, let us assume that the set **Y** of our *p* morphometric descriptors **Y**=[*Y*_1_,…,*Y_p_*], measured on the *i*-th subject/animal (our experimental unit), belonging to the *j*-th population, to be referred to the *l*-th type of cell (Basket=B, Stellate=S, Purkinje=P, Granules=Gr, Golgi=Go) located at a given ROI − region of interest (external, Purkinje, granular layer) or, can be modelled as

*Y_iljk_* = µ + τ*_ljk_* + ε*_iljk_*, (1)

where ε*_iljk_* are i.i.d. possibly non-Gaussian error terms with null mean and unknown distribution *P****_ε_***, µ is a population-invariant constant, coefficients **τ***_j_* represent the *main population effects,* and *σ*^2^(τ*_j_*) are population-varying scale coefficients which may depend, through monotonic functions, on main treatment effects **τ***_j_*. Basically, the proposed data representation model is a quite general less-demanding nonparametric model where specific location and scale effects are both allowed across populations.

Since the study’s main goal is to compare the bovine populations, we are inferring on the vector of *main population* coefficients **τ***_j_*. In order to keep a flexible and reliable inferential analysis, we are advised to apply a nonparametric testing approach; in this connection we propose a suitable extension to model (1) of the nonparametric combination NPC testing method (Bonnini et al., 2014).

For this goal, let us formalize the comparison between the *j*-th and the *h*-th population, separately for the location and scatter parameters, and for a given type of cell, via the Roy’s Union-Intersection testing approach (Pesarin and Salmaso, 2010), let use stating the null and alternative hypotheses as follow

$\left\{ \begin{matrix} H_{0\left( ljh \right)}^{loc}:\bigcap_{k}Y_{ljk}{}_{=}^{loc}{Y_{lhk}}\equiv\bigcap_{k}\left[ \tau_{ljk}=\tau_{lhk} \right] \\ \begin{matrix} \\ H_{1\left( ljh \right)}^{loc}:\bigcup_{k}\left[ \left( Y_{ljk}{}_{<}^{loc}{Y_{lhk}} \right)\bigcup\left( Y_{ljk}{}_{>}^{loc}{Y_{lhk}} \right) \right] \end{matrix} \\ \equiv\bigcup_{k}\left[ \left( \tau_{ljk}<\tau_{lhk} \right)\bigcup\left( \tau_{ljk}>\tau_{lhk} \right) \right] \end{matrix} \right.$ $\left\{ \begin{matrix} H_{0\left( ljh \right)}^{sca}:\bigcap_{k}Y_{ljk}{}_{=}^{sca}{Y_{lhk}}\equiv\bigcap_{k}\left[ \sigma_{ljk}^{2}=\sigma_{lhk}^{2} \right] \\ \begin{matrix} \\ H_{1\left( ljh \right)}^{sca}:\bigcup_{k}\left[ \left( Y_{ljk}{}_{<}^{sca}{Y_{lhk}} \right)\bigcup\left( Y_{ljk}{}_{>}^{sca}{Y_{lhk}} \right) \right] \end{matrix} \\ \equiv\bigcup_{k}\left[ \left( \sigma_{ljk}^{2}<\sigma_{lhk}^{2} \right)\bigcup\left( \sigma_{ljk}^{2}>\sigma_{lhk}^{2} \right) \right] \end{matrix} \right.$ (2)

where *l* = 1,…,5, is the reference index for the type of cell of interest.

It is worth noting that hypotheses (2) refers to a stratified version of the so-called generalized Beherens-Fisher problem (Yanagihara and Yuan, 2005), which is actually a quite difficult testing problem to handle within the traditional parametric methods (Pesarin and Salmaso, 2010). Under the null hypothesis our observed data can be considered approximately exchangeable random components that can be permuted between groups in order to derive, separately for the location and scatter problems, two combined over all morphometric features directional *p*-values.

As univariate location and scatter test statistic we respectively used the differences of sample means and squared deviations together with, as combing function to derive overall the *p*-values across all morphometric descriptors, the Fisher’s combining function. For a more in depth understanding of the testing procedure we shortly sketched here we refer the readers to Corain and Salmaso (2015).

By exploiting the combined across morphometric descriptors one-sided alternatives in expression (2), we may derive two sorts of location and scatter rankings, by using the ranking methodology proposed by Arboretti et al. (2014). In fact, by suitable combining information from directional *p*-values, the underline latent ordering among τ*_lj_* and *σ_lj_*^2^ parameters can be properly estimated. In a nutshell, the rationale behind the ranking within a multivariate setting is the following: if not all $H_{0(ljh)}$ in (2) are true, it must exist an ordering [1],[2],…,[*C*] among τ*_lj_* and *σ_lj_*^2^ such that

τ*_l_* _[1]_≤τ*_l_* _[2]_≤…≤τ*_l_* _[_*_C_*_]_ and *σ* ^2^*_l_* _[1]_≤*σ* ^2^*_l_* _[2]_≤…≤*σ* ^2^*_l_* _[_*_C_*_]_,

where *C* is the number of populations to be ranked (3 in our case).

For more details on the ranking methodology we refer the reader to Arboretti et al. (2014), and Corain et al. (2016).
